# Getting unstuck from a stagnant career: examining the role of perceived mentor proactivity in the relationship between protégés career plateaus and workplace avoidance behavior

**DOI:** 10.3389/fpsyg.2026.1920144

**Published:** 2026-08-25

**Authors:** Yufu Wang, Muhammad Zada, Hammad S. Alotaibi, Alejandro Vega-Muñoz, Nicolás Contreras-Barraza, Jawad Khan, Kishwar Ali

**Affiliations:** 1 School of Management, Jiangsu University, Zhenjiang, China; 2 School of Economics and Management, Hanjiang Normal University Shiyan, Shiyan, China; 3 Facultad de Administración y Negocios, Universidad Autónoma de Chile, Santiago, Chile; 4 College of Taraba, Taif University, Taif, Saudi Arabia; 5 Facultad de Medicina y Ciencias de la Salud, Universidad Central de Chile, Santiago, Chile; 6 Facultad de Ciencias Empresariales, Universidad Arturo Prat, Iquique, Chile; 7 Facultad de Ciencias Económicas y Administrativas, Pontificia Universidad Católica de Valparaíso, Valparaíso, Chile; 8 ARUCAD Research Centre, Arkin University of Creative Arts and Design, Northern Cyprus, Mersin, Northern Cyprus, Türkiye; 9 Advanced Research Centre, European University of Lefke, Mersin, Northern Cyprus, Türkiye

**Keywords:** affective events theory, career plateaus, job frustration, perceived mentor proactivity, workplace avoidance behavior

## Abstract

Drawing on affective events theory, this study examines how protégés’ career plateaus influence workplace avoidance behavior through job frustration and how perceived mentor proactivity shapes this process. Using a three-wave, time-lagged, multi-source design with data collected from 401 mentor–protégé dyads in the service sector, the findings show that both hierarchical plateauing and job-content plateauing increase protégés’ job frustration, which subsequently promotes workplace avoidance behavior. The results further reveal that perceived mentor proactivity weakens the positive relationships between protégés’ career plateaus and job frustration. In addition, perceived mentor proactivity attenuates the indirect effects of hierarchical and job-content plateauing on workplace avoidance behavior through job frustration. These findings extend the career plateauing and workplace avoidance literature by explaining how career stagnation produces negative affective reactions that translate into avoidance-oriented behavior. The study also highlights the protective role of proactive mentors in reducing protégés’ frustration and preventing adverse workplace outcomes. Practical implications are offered for organisations seeking to manage career plateauing, strengthen mentoring effectiveness, and reduce workplace avoidance among protégés.

## Introduction

The contemporary career landscape is marked by instability, uncertainty, and rapid change due to economic fluctuations, technological advancement, and workforce diversity (Ng and Yang, 2023). In this environment, career growth remains an important source of motivation, yet flatter organizational structures and restricted promotion opportunities increasingly expose protégés to career plateauing (e.g., Allen et al., 2004; Wei and Yu, 2023). Career plateauing refers to a perceived ceiling in career advancement, reflected in limited promotion prospects (hierarchical plateau) or insufficient challenge and responsibility in the current role (job-content plateau) (Hu et al., 2022). Both forms can undermine protégés’ sense of career worth and purpose (Kao et al., 2022) and have been associated with lower career commitment, job satisfaction, organizational commitment, and well-being, as well as stronger turnover intentions (Hofstetter and Cohen, 2014; Lin and Chen, 2021; Yang et al., 2019). However, this literature primarily explains how plateaued protégés’evaluate their jobs or contemplate leaving, offering less insight into how they regulate their everyday participation when they remain in the organization.

We address this gap by examining workplace avoidance behavior, which reflects a pattern of reducing one’s physical, cognitive, and temporal involvement in work through absenteeism, disengagement, and procrastination (Iqbal et al., 2024; Ng et al., 2016). Workplace avoidance behavior is related to, but conceptually distinct from, outcomes commonly examined in career plateau research. Absenteeism and procrastination are specific acts, disengagement captures diminished involvement, and turnover intention concerns a contemplated future exit (Grifno et al., 2025; Hen et al., 2021; Metin et al., 2016). Withdrawal is a broader category of distancing responses, whereas counterproductive work behavior also includes active acts intended to violate organizational norms or harm organizational interests (Yang et al., 2025). Workplace avoidance behavior captures the shared evasive orientation across reduced presence, attention, and timely task completion and can occur without an intention to quit or cause harm (Johnson et al., 2026). This distinction is important because plateaued protégés may stay owing to limited alternatives while progressively withholding effort from their existing roles. Such avoidance can be difficult to detect, yet its cumulative effects can disrupt coordination, delay work, and weaken dependable task execution. Although career plateauing has been connected to counterproductive tendencies (Jain and Chhabra, 2024; Ng and Yang, 2023), its relationship with this quieter, within-employment pattern of avoidance remains insufficiently understood.

Given the untapped nature of the inquiry, this study contends that the relationship between protégés career plateaus and workplace avoidance behavior may not be straightforward. Instead, there may be an underlying mechanism through which protégés’ career plateaus transform their influence on workplace avoidance behavior. Drawing on affective event theory (Weiss and Cropanzano, 1996), we propose job frustration as the affective mechanism linking career plateauing to workplace avoidance behavior. Career plateauing signals that valued goals involving advancement, learning, or responsibility have been obstructed. Job frustration—the negative affect arising when organizational or personal conditions block goal attainment (Vander Elst et al., 2012)—captures the immediate emotional significance of this obstruction. This mechanism adds theoretical insight by separating the career condition from the emotion that translates it into behavior (Crossman et al., 2009; Zhang et al., 2011; Zia et al., 2025). Career plateauing therefore does not mechanically produce avoidance; it generates frustration that encourages protégés to distance themselves from work activities associated with blocked aspirations. AET thus explains why avoidance, rather than only an unfavorable attitude or an intention to leave, may emerge from career stagnation.

This study considers the construct of perceived mentor proactivity, which refers to a protégé’s subjective evaluation of their mentor’s self-directed initiative in guiding, supporting, and creating growth opportunities (Wang et al., 2014), as a boundary condition. This construct differs from mentor support, supervisor support, mentoring quality, and perceived support, which primarily describe the availability, receipt, or evaluation of assistance. A proactive mentor is inclined to act before assistance is requested by initiating career conversations, identifying developmental assignments, expanding professional connections, and helping protégés construct alternative routes for growth (Kim and Beehr, 2018; Ragins et al., 2024). From an AET perspective, these anticipatory actions can change how a plateau is interpreted by making viable career possibilities more salient, thereby weakening the frustration generated by perceived stagnation. Recent evidence indicates that proactive career support reduces perceptions of job-content plateauing, while plateauing produces negative emotions that can translate into adverse workplace behavior (Jiang et al., 2025; Ng and Yang, 2024). The mentor’s proactivity therefore provides a person-based boundary condition explaining why protégés facing similar plateaus may experience different emotional and behavioral consequences. This boundary condition is particularly important because the formal availability of mentoring does not guarantee equivalent developmental assistance; mentors differ in their interest, career guidance, and relationship quality (Ragins et al., 2024).

**Figure 1:**
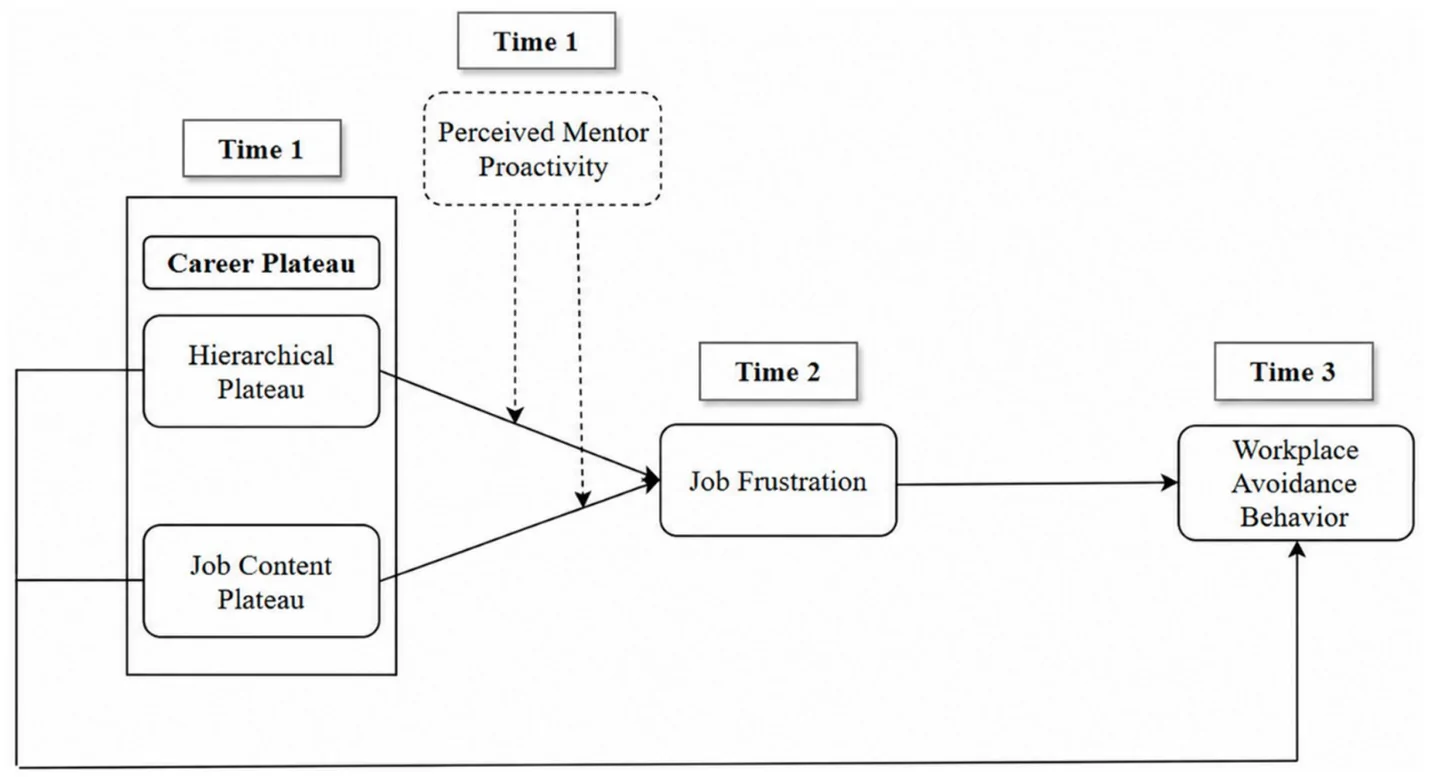
Theoretical model.

This study makes three contributions. First, it extends career plateau research beyond its traditional emphasis on unfavorable work attitudes, turnover intentions, and broadly defined counterproductive work behavior by examining workplace avoidance behavior. Workplace avoidance behavior captures how plateaued protégés may remain employed while reducing their physical, cognitive, and temporal participation through absenteeism, disengagement, and procrastination. This focus reveals a subtle but operationally important consequence of career plateauing that differs from contemplating departure, exhibiting a single withdrawal behavior, or deliberately engaging in harmful workplace conduct. Second, the study advances AET by explaining the affective process through which career plateauing produces avoidance behavior. Job frustration is conceptualized as the proximal emotional response to the obstruction of valued career goals, rather than simply another unfavorable job attitude. Its mediating role distinguishes the plateau as a work event from the emotional reaction that translates perceived stagnation into behavioral distancing, thereby explaining why hierarchical and job-content plateaus may generate workplace avoidance. Third, this study introduces perceived mentor proactivity as an interpersonal boundary condition that explains why protégés experiencing similar career plateaus may respond differently. When mentors are perceived as anticipating career obstacles and initiating developmental solutions, protégés are more likely to recognize alternative growth paths and view career stagnation as manageable. Perceived mentor proactivity therefore clarifies when mentoring relationships weaken the effects of career plateauing on job frustration and subsequent workplace avoidance behavior. The proposed model is shown in Figure 1.

## Literature review and hypotheses development

### Hierarchical plateau, job content plateau, and workplace avoidance behavior

Career plateauing is a stage in which a protégé perceives low chances of future promotion. This stage is typically undesirable, as plateaued protégés often find themselves in a stable, unchanging role, performing repetitive tasks with slight variation (e.g., Jain and Chhabra, 2024; Ng and Yang, 2023). Career plateaus can be categorized as hierarchical and job-content plateaus. Hierarchical plateauing refers to employees facing limited opportunities for upward mobility within the organizational hierarchy. In contrast, job content plateauing occurs when an individual feels a lack of challenge or growth in their current position, with no new responsibilities to take on (e.g., Lin and Chen, 2021; Wang et al., 2014).

Experiencing a career plateau can lead to various adverse outcomes that affect an employee’s sense of self-worth, perceived contribution to the organization, and overall career purpose. These effects may contribute to situations where plateaued protégés intentionally avoid completing tasks or responsibilities related to their job, as employees who feel stagnant or undervalued may begin to disengage (e.g., Jain and Chhabra, 2024; Ng and Yang, 2023). Plateaued protégés’ disengagement can manifest as physical or psychological avoidance, where protégés minimize their presence or emotional investment in their roles to cope with feelings of frustration, insignificance, or lack of progress (Tremblay, 2021). When career growth and challenging responsibilities are absent, the motivation to actively participate and contribute can decline, leading protégés to withdraw or limit their involvement in the workplace (Yang et al., 2018). The constraints of a hierarchical plateau become particularly apparent as protégés perceive a ceiling on their advancement within the organization. This stage can be challenging and stressful, as limited growth opportunities affect overall work attitudes (Xie et al., 2016).

According to the affective events theory (Wegge et al., 2006), experiencing a hierarchical plateau can be an emotional trigger, leading protégés to feel undervalued and disconnected from their roles. This sense of disconnection often leads protégés to disengage from their work, reducing their involvement in tasks and interactions (e.g., De Clercq et al., 2020; McCleese et al., 2007). Without opportunities for feedback or career progression, protégés who plateau may struggle to adapt to organizational demands or meaningfully cope with challenges (Nachbagauer and Riedl, 2002). As a result, they may withdraw from situations where their efforts seem unrecognized or ineffective, using this withdrawal to manage the emotional strain of their plateaued position (Ferris et al., 2016).

According to Hu et al. (2022), protégés often feel plateaued when their work becomes monotonous and lacks challenge or meaning. In such situations, they may become less motivated to engage with their tasks or invest in improving their performance. A job content plateau can arise internally when protégés stop learning and acquiring new skills, eventually realizing they have outgrown their current role yet lack the necessary skills for advancement. Externally, it can occur when employers fail to recognize or value plateaued protégés expertise and experience (e.g., Huo and Jiang, 2023; Hurst et al., 2017). From the perspective of affective events theory, these situations trigger negative emotional responses in protégés, leading to a perceived loss of resources at work (Lin and Chen, 2021). As a result, protégés may feel disconnected from their roles and interests, leading them to disengage from their work (Hofstetter and Cohen, 2014). This emotional disconnection may cause them to withdraw from their tasks and limit their involvement in the workplace, as they feel less motivated and less identified with their job responsibilities (Hershcovis et al., 2018; Ng et al., 2016). The lack of challenge and recognition can foster an environment where protégés resist further career disruption and begin to avoid situations that highlight their stagnation (Akkermans et al., 2021). Thus, we hypothesize the following:

*Hypothesis 1:* Protégés hierarchical plateau is positively related to workplace avoidance behavior

*Hypothesis 2:* Protégés job content plateau is positively related to workplace avoidance behavior

### The mediating role of job frustration in the relation of hierarchical and job content plateau to workplace avoidance behavior

Job frustration is a negative emotion triggered by adverse workplace events (Zhang et al., 2011). In the workplace, job frustration occurs when protégés encounter persistent barriers or lack opportunities for growth (Lin and Chen, 2021). Affective events theory (Wegge et al., 2006) explains how workplace events, such as career plateauing, trigger emotional responses that influence subsequent behaviors. In this context, career plateauing is a potential stressor leading to adverse emotional reactions, such as job frustration (Tremblay, 2021). When protégés experience frustration due to perceived stagnation in their roles—whether from a lack of advancement (hierarchical plateau) or a lack of challenge (job content plateau)—it can elicit uncomfortable emotional responses (e.g., Hu et al., 2022; Nachbagauer and Riedl, 2002).

In line with AET, negative emotions can drive protégés to avoid tasks or responsibilities at work and spend too much time on non-work activities to cope with or alleviate the negative feelings associated with their plateaued state (e.g., DiRenzo et al., 2022; Lin et al., 2018). According to Chin et al. (2022), plateaued protégés who perceive their stagnant state as a threat are more likely to experience emotional exhaustion and dissatisfaction stemming from unmet career expectations and, in turn, intentionally avoid completing tasks or responsibilities to manage the discomfort caused by their feelings of stagnation. Wickramasinghe and Jayaweera (2010) argued that hierarchical and job content plateaus—situations where protégés feel constrained in career advancement or experience monotony in their roles—do more than simply block goal achievement and contribute to a sense of frustration, a state of emotional discomfort that arises when employees perceive barriers to personal growth, skill development, or meaningful contribution (Chang Boon Lee, 2003). Protégés who feel unchallenged or unable to advance may withdraw effort or disengage from work activities to manage these negative feelings, or they may avoid tasks they see as perpetuating their dissatisfaction (e.g., Jain and Chhabra, 2024; Smith-Ruig, 2009). In a workplace context, frustration significantly shapes deliberate behavioral responses, such as avoiding work tasks or environments (Crossman et al., 2009). Protégés may use avoidance as a retaliatory response to the perceived source of frustration (Spector and Fox, 2002). Moreover, frustrated protégés expend effort to manage their negative emotions, depleting their self-regulatory resources and reducing self-control. This regulatory depletion, in turn, heightens the likelihood of engaging in more self-centered and antisocial behaviors (e.g., Gao et al., 2024; Oglensky, 2008).

We argue that when protégé feel trapped in their current roles with no prospects for advancement or challenge, they are likely to experience job frustration, which leads them to withdraw from their tasks and reduce their engagement in the workplace. We propose the following hypothesis:

*Hypothesis 3a:* Job frustration mediates the relationship between protégés' hierarchical plateau and workplace avoidance behavior

*Hypothesis 3b:* Job frustration mediates the relationship between protégés' job content plateau and workplace avoidance behavior

### Perceived mentor proactivity as a moderator

According to Wang et al. (2014), mentoring is a critical resource investment that helps protégés navigate career plateaus. Proactive mentors support their protégés’ career development by identifying growth opportunities, taking initiative, and actively addressing career challenges (Jena et al., 2024). Such mentors persist alongside their protégés, fostering progress and enabling them to achieve new career milestones (Wei and Yu, 2023). According to Baral et al. (2024), mentors help protégés recognize new avenues for growth, overcome stagnation, and navigate hierarchical and job content plateaus. With this support, protégés are more likely to pursue opportunities for career growth and take proactive steps, thereby reducing feelings of frustration (e.g., Wang et al., 2014; Zeng et al., 2020).

From the perspective of affective event theory (Weiss and Cropanzano, 1996), workplace events like career plateaus can evoke negative emotional responses, such as frustration, due to stagnation or limited advancement opportunities (Unnikrishnan and Rajeev, 2024). Perceived mentor proactivity can mitigate these negative emotions by providing guidance, emotional support, and resources, which foster positive coping strategies and reduce job frustration (Liu et al., 2024). Protégés often rely on mentors as vital sources of career support during challenging periods, including career plateaus (Joo and Cruz, 2024). With the encouragement of proactive mentors, protégés are more likely to identify growth opportunities independently, seek meaningful changes, and promote self-development. This reduces emotional exhaustion and dissatisfaction from unmet career expectations (Liu et al., 2024). Therefore, we propose that perceived mentor proactivity can buffer the adverse effects of hierarchical and job content plateaus on protégés’ job frustration.

*Hypothesis 4a:* The positive relationship between protégés' hierarchical plateau and job frustration is moderated by the perceived mentor proactivity, such that the relationship is weak when the perceived mentor proactivity is high (vs. low).

*Hypothesis 4b:* The perceived mentor proactivity moderates the positive relationship between protégés' job content plateau and job frustration, such that the relationship is weak when the perceived mentor proactivity is high (vs. low).

### The moderated mediation model

Integrating hypotheses 3a, 3b, 4a, and 4b, we predict that protégés’ career plateaus (i.e., hierarchical and job content plateaus) indirectly influence workplace avoidance behavior through job frustration. However, for protégés, the relationship between career plateaus, job frustration, and increased workplace avoidance behavior is weaker when the perceived mentor proactivity is high. We anticipate that, under mentor proactivity, the impact of career plateaus on job frustration is diminished, thereby reducing the positive indirect effect on workplace avoidance behavior.

*Hypothesis 5a:* The indirect relationship of a hierarchical plateau with workplace avoidance behavior via job frustration will be moderated by perceived mentor proactivity. Specifically, the indirect relationship will be weaker when perceived mentor proactivity is high and stronger when perceived mentor proactivity is weak.

*Hypothesis 5b:* The perceived mentor proactivity will moderate the indirect relationship of job content plateau with workplace avoidance behavior via job frustration. Specifically, the indirect relationship will be weaker when perceived mentor proactivity is high and stronger when perceived mentor proactivity is weak.

## Methods

### Sample and procedure

We collected data from formal mentor–protégé dyads employed in service-sector organizations in China. The dyads were formed based on formal mentoring relationships established within the participating organizations. In each dyad, the mentor was also the protégé’s direct supervisor and had been formally designated to provide career guidance and developmental support. Participants were drawn from the hospitality industry (32%), banking and financial services (22%), information technology services (18%), telecommunications (12%), retail services (10%), and professional services (6%), including legal consulting, HR outsourcing, marketing agencies, and business consultancy firms. Each mentor was matched with one formally assigned protégé and evaluated only that protégé. Participation was voluntary, and written informed consent was obtained from both members of each dyad.

Data were collected across three waves separated by two-month intervals to reduce concerns about common method bias and establish temporal separation among the focal variables (Podsakoff et al., 2012). At Time 1, protégés reported their hierarchical plateau, job-content plateau, perceived mentor proactivity, and demographic information. Two months later, the same protégés completed the Time 2 survey assessing job frustration. At Time 3, 2 months after Time 2, mentors, acting in their supervisory capacity, rated their protégés’ workplace avoidance behavior. This multi-wave and multi-source design reduced the likelihood that the observed relationships were inflated by common method variance (Podsakoff et al., 2003).

At Time 1, 600 questionnaires were distributed to protégés, of which 546 were completed and returned. After four questionnaires containing missing information were excluded, 542 valid responses remained. At Time 2, these protégés were invited to participate again, yielding 411 returned questionnaires. Four incomplete questionnaires were removed, leaving 407 valid responses. At Time 3, questionnaires were distributed to the formal mentors of the 407 protégés who had completed the Time 2 survey. To match responses across the three waves and between the two members of each dyad, every participant was assigned a unique anonymised identification code at Time 1. The same code was used across all survey waves and on both the mentor and protégé questionnaires. After six incomplete or unmatched responses were excluded, the final sample comprised 401 matched mentor–protégé dyads. The final matched response rate was 66.8%, representing an attrition rate of approximately 33.2% across the three waves. Among the protégés, 60.10% were male and 39.90% were female. Their average age was 33.73 years, and their average work experience was 3.05 years. Regarding education, 69% held a master’s degree or higher.

### Common method bias

To address common method bias, we employed a time-lagged design with a two-month interval between consecutive rounds of data collection and data from two sources. Prior research suggests that a two-month interval is effective, as it strikes a balance between being long enough to reduce recall effects and short enough to minimize the influence of extraneous factors (Usman et al., 2024). Unique identification codes were used to link participants’ responses across the different survey waves and maintain consistency in the matched data. We also assessed the possibility of common method bias by applying a confirmatory factor analysis marker-variable approach (Williams et al., 2010). The study utilized attitudes toward the color blue as a marker variable, selected for its theoretical independence from the core variables under investigation. Drawing on the approach described by Simmering et al. (2015), these attitudes were evaluated through a three-item scale comprising the statements: “I like the color blue,” “I prefer blue over other colors,” and “I like blue clothing” (*α* = 0.88). In conducting the CFA with the marker variable, we specified three model configurations: an unconstrained model, a fully constrained model (fixed at zero to ensure no shared variance with the marker variable), and an equally constrained model. We compared the chi-square (*χ*^2^) and degrees of freedom (*df*) of the unconstrained and fully constrained models to assess potential method bias. The chi-square difference test (Δ*χ*^2^ = 42.81, *df* = 42, *p* = 0.51) indicated no significant shared variance due to response bias, suggesting minimal method bias. Furthermore, the equally constrained model revealed that less than 1% of the variance was shared between the primary constructs and the marker variable, supporting the robustness of our findings.

### Measures

#### Career plateauing

Career plateauing was assessed through two dimensions: hierarchical plateauing and job-content plateauing. Each dimension was measured with six items adapted from Milliman (1993). Samples are “I am unlikely to obtain a much higher job title in my organization” (hierarchical plateauing) and “My job tasks and activities will become routine for me in the future” (job content plateauing).

#### Job frustration

Job frustration was assessed with a three-item measure developed by Peters et al. (1980). A sample item is “trying to get this ‘job’ done was a very frustrating experience.”

#### Perceived mentor proactivity

We measured the perceived mentor proactivity using the six-item scale, adapted from the shortened version (Parker, 1998) from the original 17 items (Bateman and Crant, 1993). An example item is, “My mentor is always looking for better ways to do things.”

#### Workplace avoidance behavior

We measure workplace avoidance behaviors by adapting a seven-item scale used in past studies (e.g., Iqbal et al., 2024; Linden et al., 2008; Muschalla and Linden, 2009). A sample item is “Whenever possible, this employee avoids approaching the workplace site.”

#### Control variables

We included age, gender, job tenure, and education as control variables because prior studies suggest that these demographic and work-related characteristics may influence job frustration and workplace avoidance behavior (Iqbal et al., 2024).

## Results

### Means and correlations

Table 1 reports the descriptive statistics and bivariate correlations for the study variables. The correlation results generally support the expected pattern of relationships among the main constructs.

**Table 1:** Means and correlations.

Variable	*M*	*SD*	1	2	3	4	5	6	7	8	9
1. Age	33.73	7.01	—								
2. Gender	1.40	0.49	0.05	—							
3. Tenure	3.05	1.39	0.02	−0.01	—						
4. Education	3.14	1.04	0.03	−0.06	−0.01	—					
5. Hierarchical Plateau	3.05	1.17	−0.10*	−0.03	−0.03	0.02	(0.92)				
6. Job-Content Plateau	3.64	1.47	0.03	0.05	−0.07	−0.05	0.38**	(0.90)			
7. Perceived Mentor Proactivity	3.27	1.31	−0.06	−0.06	0.02	0.02	−0.20**	−0.11*	(0.95)		
8. Job Frustration	3.22	1.30	0.03	0.03	−0.04	−0.05	0.19**	0.16**	−0.21**	(0.86)	
9. Workplace Avoidance Behavior	2.90	1.36	0.04	−0.07	−0.01	0.02	0.31**	0.28**	−0.11*	0.25**	(0.91)

*N* = 401, *p < 0.05. **p < 0.01. Values in parentheses on the diagonal are Cronbach’s alpha coefficients.

### Measurement model

We assessed the measurement model through confirmatory factor analysis using Mplus 8.6. The hypothesised five-factor model demonstrated a good fit to the data: *χ*^2^(340) = 380.09, *χ*^2^/*df* = 1.12, RMSEA = 0.02, SRMR = 0.03, CFI = 0.95. All standardised factor loadings exceeded 0.60 and were statistically significant (see Table 2).

**Table 2:** Confirmatory factor analysis of discriminant validity^a.^

Model	Factors	*χ* ^2^	*df*	*χ* ^2^ */df*	*Δχ* ^2^	*RMSEA*	*SRMR*	*CFI*
M 1	5 Factors: HP, JCP, PMP, JF, WAB	380.09	340	1.12	—	0.02	0.03	0.95
M 2	3 Factors: HP + JCP; PMP; JF + WAB	1958.88	347	5.65	1578.79***	0.11	0.14	0.78
M 3	1 Factor: HP + JCP + PMP + JF + WAB	5499.79	350	15.71	5119.70***	0.19	0.21	0.31

^a^M, Model, HP; Hierarchical Plateau; JCP, Job Content Plateau; PMP, Perceived Mentor Proactivity; JF, Job Frustration; WAB, Workplace Avoidance Behavior.

### Hypotheses testing

We tested the hypotheses using observed-variable structural equation modelling in Mplus 8.6 with maximum-likelihood estimation. Before forming the interaction terms, all focal predictors were grand-mean centered. Indirect effects, conditional indirect effects, and indices of moderated mediation were evaluated using 5,000 bootstrap resamples with 95% percentile-bootstrap confidence intervals. An effect was considered significant when its confidence interval excluded zero. Simple slopes were estimated at low (−1 SD) and high (+1 SD) levels of perceived mentor proactivity.

As reported in Table 3, hierarchical plateauing was positively associated with workplace avoidance behavior (*B* = 0.32***, *SE* = 0.06, 95% CI [0.21, 0.43]). Job-content plateauing was also positively associated with workplace avoidance behavior (*B* = 0.23***, *SE* = 0.04, 95% CI [0.14, 0.31]). Therefore, Hypotheses 1 and 2 were supported. Hierarchical plateauing was positively associated with job frustration (*B* = 0.21***, *SE* = 0.05, 95% CI [0.10, 0.32]), as was job-content plateauing (*B* = 0.14**, *SE* = 0.04, 95% CI [0.06, 0.23]). Job frustration, in turn, was positively related to workplace avoidance behavior (*B* = 0.21***, *SE* = 0.05, 95% CI [0.11, 0.31]). The indirect effect of hierarchical plateauing on workplace avoidance behavior through job frustration was significant (*B* = 0.04*, *SE* = 0.02, 95% CI [0.02, 0.08]). The corresponding indirect effect of job-content plateauing was also significant (*B* = 0.03*, *SE* = 0.01, 95% CI [0.01, 0.06]), thus Hypotheses 3a and 3b were supported.

**Table 3:** Hypotheses results.

Constructs	Job frustration			Workplace avoidance behavior		
	*B*	*SE*	95% CI	*B*	*SE*	95% CI
Age	0.01	0.01	[−0.01, 0.02]	0.01	0.01	[−0.01, 0.03]
Gender	0.05	0.11	[−0.16, 0.27]	−0.22	0.13	[−0.47, 0.03]
Education	−0.01	0.05	[−0.11, 0.09]	0.04	0.06	[−0.08, 0.16]
Job tenure	−0.02	0.04	[−0.09, 0.06]	0.01	0.05	[−0.07, 0.10]
Hierarchical plateau	0.21***	0.05	[0.10, 0.32]	0.32***	0.06	[0.21, 0.43]
Job-content plateau	0.14**	0.04	[0.06, 0.23]	0.23***	0.04	[0.14, 0.31]
Job frustration				0.21***	0.05	[0.11, 0.31]
Interactions						
Hierarchical plateau × perceived mentor proactivity	−0.34***	0.04	[−0.42, −0.27]			
Job-content plateau × perceived mentor proactivity	−0.30***	0.03	[−0.36, −0.24]			
Indirect effects						
Hierarchical plateau → job frustration → Workplace avoidance behavior				0.04*	0.02	[0.02, 0.08]
Job-content plateau → job frustration → Workplace avoidance behavior				0.03*	0.01	[0.01, 0.06]
Moderated mediation						
Index of moderated mediation for hierarchical plateau				−0.07*	0.02	[−0.11, −0.04]
Index of moderated mediation for job-content plateau				−0.06*	0.02	[−0.10, −0.04]
Simple slope analysis						
Hierarchical plateau → Job frustration at high perceived mentor proactivity (+1 SD)	−0.28***	0.07	[−0.42, −0.14]			
Hierarchical plateau → Job frustration at low perceived mentor proactivity (−1 SD)	0.62***	0.07	[0.49, 0.76]			
Job-content plateau → Job frustration at high perceived mentor proactivity (+1 SD)	−0.23***	0.05	[−0.33, −0.13]			
Job-content plateau → Job frustration at low perceived mentor proactivity (−1 SD)	0.56***	0.06	[0.44, 0.67]			

*N* = 401. *p < 0.05. **p < 0.01. ***p < 0.001.

**Figure 2:**
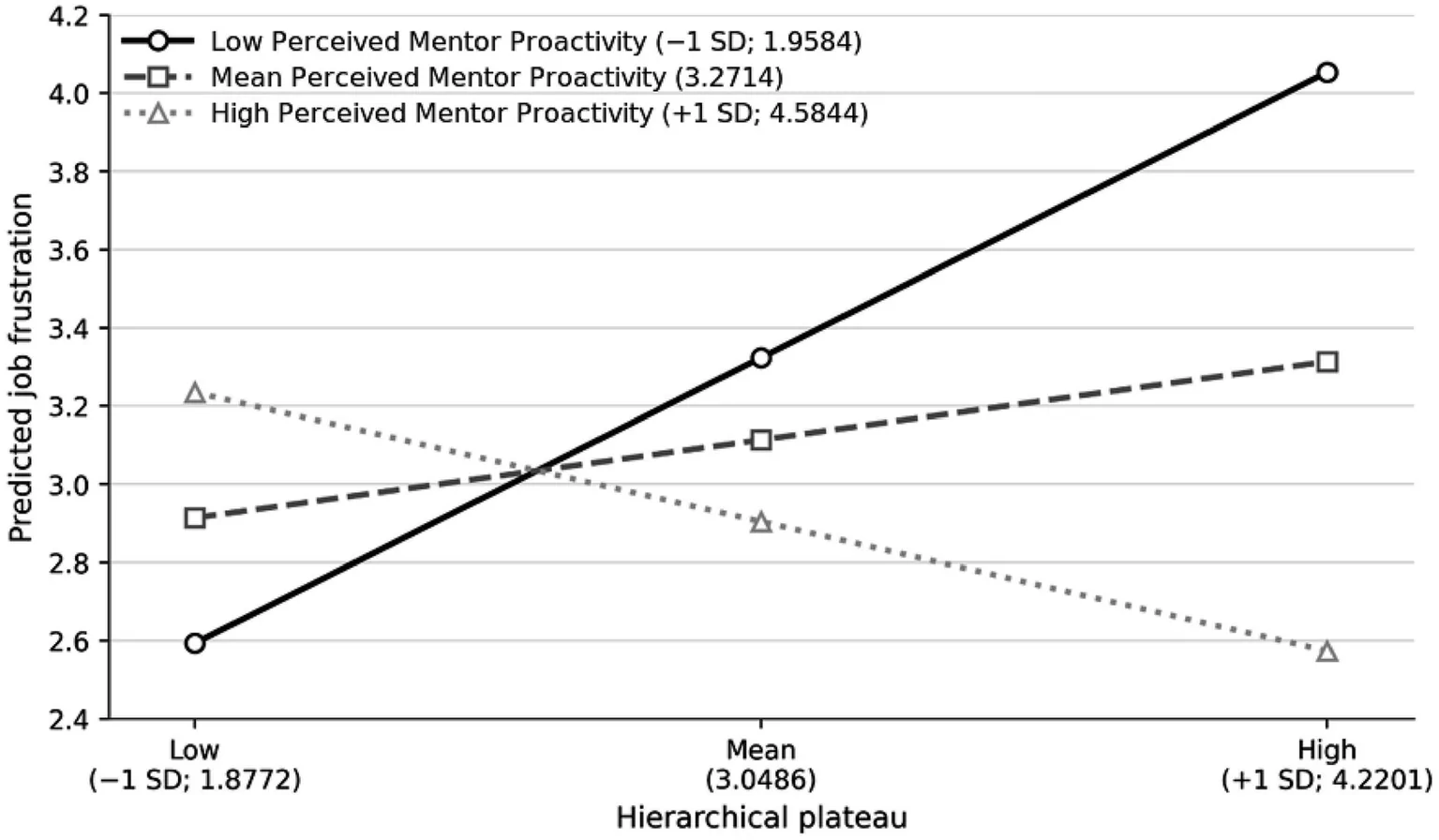
Interaction of hierarchical plateau and perceived mentor proactivity predicting job frustration.

**Figure 3:**
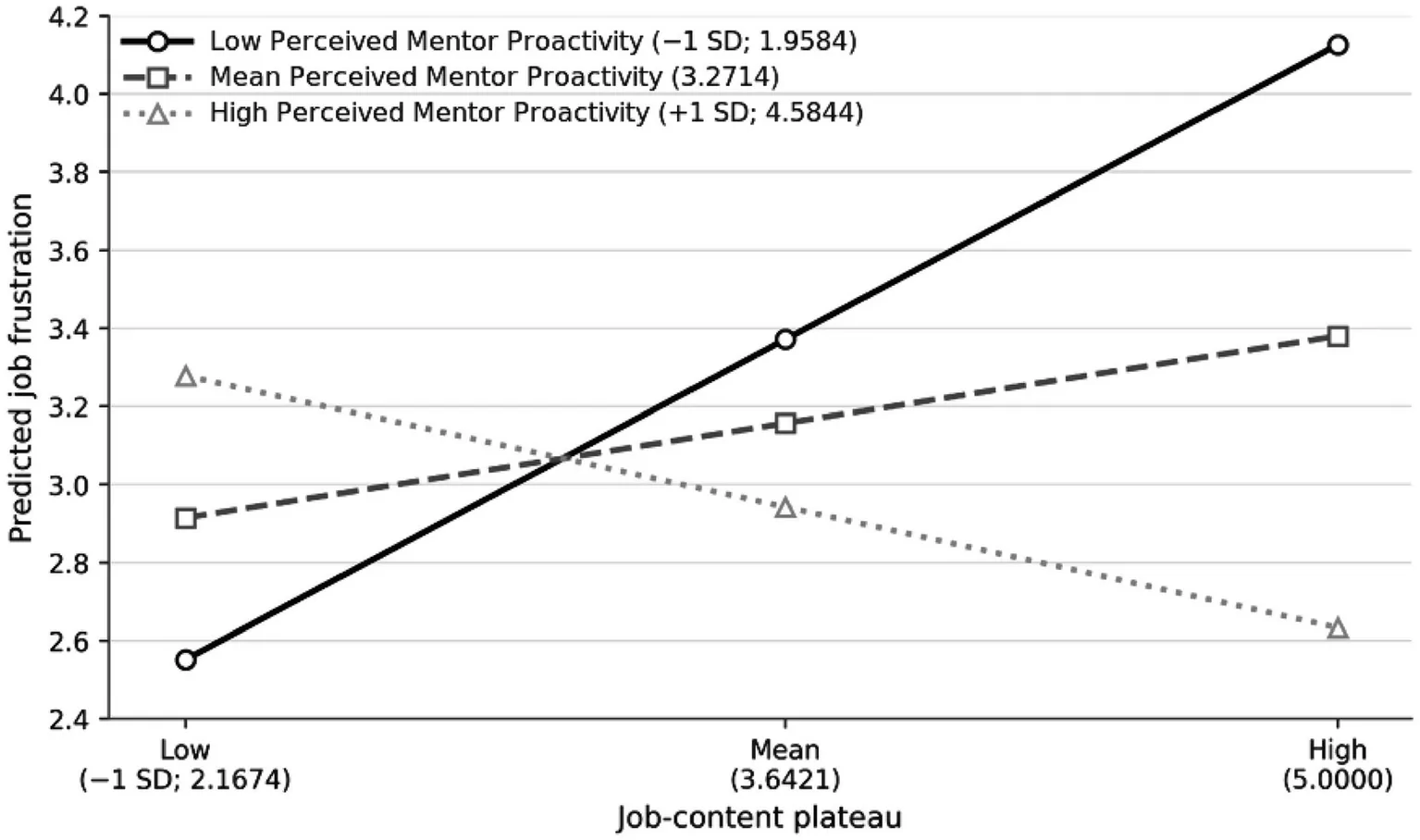
Interaction of job-content plateau and perceived mentor proactivity predicting job frustration.

Perceived mentor proactivity significantly moderated the relationship between hierarchical plateauing and job frustration (*B* = −0.34***, *SE* = 0.04, 95% CI [−0.42, −0.27]). At low perceived mentor proactivity (−1 SD), hierarchical plateauing was positively associated with job frustration (*B* = 0.62***, *SE* = 0.07, 95% CI [0.49, 0.76]). At high perceived mentor proactivity (+1 SD), the relationship became negative (*B* = −0.28***, *SE* = 0.07, 95% CI [−0.42, −0.14]) (see Figure 2). The interaction between job-content plateauing and perceived mentor proactivity was also negative and significant (*B* = −0.30***, *SE* = 0.03, 95% CI [−0.36, −0.24]). At low perceived mentor proactivity, job-content plateauing was positively associated with job frustration (*B* = 0.56***, *SE* = 0.06, 95% CI [0.44, 0.67]). At high perceived mentor proactivity, the relationship became negative (*B* = −0.23***, *SE* = 0.05, 95% CI [−0.33, −0.13]) (see Figure 3). These findings support Hypotheses 4a and 4b. These negative slopes indicate crossover interactions rather than simple attenuation. At high perceived mentor proactivity, both hierarchical plateauing (*B* = −0.28***) and job-content plateauing (*B* = −0.23***) were associated with lower job frustration. This pattern does not imply that career plateauing becomes beneficial. Rather, it suggests a particularly strong buffering association, possibly because proactive mentors help protégés identify alternative growth pathways and view stagnation as manageable.

The index of moderated mediation for hierarchical plateauing was negative and significant (index = −0.07*, *SE* = 0.02, 95% CI [−0.11, −0.04]). This finding indicates that the indirect relationship between hierarchical plateauing and workplace avoidance behavior through job frustration decreased as perceived mentor proactivity increased. The index of moderated mediation for job-content plateauing was also negative and significant (index = −0.06*, *SE* = 0.02, 95% CI [−0.10, −0.04]). Thus, the indirect relationship between job-content plateauing and workplace avoidance behavior through job frustration similarly weakened as perceived mentor proactivity increased. Because neither bootstrap confidence interval included zero, Hypotheses 5a and 5b were supported.

## Discussion

### Theoretical implications

This study makes several significant theoretical contributions to understanding career plateauing, particularly by linking it to job frustration and workplace avoidance behavior. It also considers the role of the perceived mentor proactivity. First, workplace avoidance behavior, a form of counterproductive work behavior (CWB), includes disengagement from job tasks, absenteeism, or withdrawal from organizational activities (Iqbal et al., 2024). Previous research has established links between career plateaus and a variety of negative work outcomes, including job dissatisfaction (Hurst et al., 2017), turnover intentions (Xie et al., 2016), and decreased organizational commitment (Nachbagauer and Riedl, 2002). The direct relationship between career plateauing and workplace avoidance behavior remains underexplored. Most studies have focused on the general emotional and motivational consequences of career plateauing (e.g., Gustafsson and Kärreman, 2023; Lin and Chen, 2021; Wang et al., 2014). This study addresses the literature gap by examining how career plateaus among protégés contribute to workplace avoidance behaviors, shedding light on how a lack of advancement opportunities may drive withdrawal tendencies in the workplace.

Second, this study identifies job frustration as an emotional reaction to career plateaus that leads to workplace avoidance behavior. While career plateauing has been recognized as an emotionally unpleasant experience, previous research has not fully explored the role of emotional responses, mainly frustration (Iqbal et al., 2024), in explaining how career plateaus influence behaviors like avoidance in the workplace. This study proposes that job frustration serves as a mediator, linking career stagnation to adverse workplace outcomes, such as disengagement and withdrawal. Examining this emotional process offers a deeper understanding of how plateauing translates into adverse behaviors, rather than assuming that plateaued employees will automatically exhibit negative outcomes (Legrand et al., 2023). This approach refines the existing literature by shifting the focus from surface-level outcomes to the underlying emotional dynamics that drive such behaviors, echoing the work of researchers like affective event theory (Weiss and Cropanzano, 1996), emphasizing the impact of emotional reactions on subsequent workplace behaviors. In doing so, this study provides a more detailed and nuanced understanding of the mechanisms through which career plateaus lead to workplace avoidance behaviors.

Furthermore, this study introduces the perceived mentor proactivity as a critical boundary condition that moderates the relationship between career plateaus and job frustration, as well as the indirect link between career plateaus and workplace avoidance behavior. The role of mentors has been recognized in the literature as crucial in career development and emotional support (e.g., Wang et al., 2014). Prior research has largely overlooked how a mentor’s proactivity may moderate the impact of career plateaus on protégé outcomes, such as job frustration and workplace avoidance behavior. By focusing on perceived mentor proactivity, this study suggests that actively engaged and supportive mentors can help mitigate the adverse effects of career plateaus (e.g., Wei and Yu, 2023).

Specifically, mentor proactivity is more likely to recognize when their protégés are struggling with plateauing and take steps to address it (e.g., Zeng et al., 2020), thereby reducing job frustration and preventing it from turning into avoidance behavior. This builds on earlier studies emphasizing the importance of mentor support (Ragins and Kram, 2007) but extends this literature by incorporating a specific trait—proactivity—that enhances the mentor’s ability to intervene effectively (Lentz and Allen, 2009). In addition to its contributions to understanding the mentor-protégé relationship, this study challenges the conventional view of plateaued employees as inherently disengaged or problematic. Previous research has often focused on career plateaus as primarily negative experiences that lead to disengagement (e.g., Ng and Yang, 2023). However, this study suggests that the emotional response to a plateau is mainly managed with proactive mentoring. This study emphasizes the importance of understanding the emotional dynamics and the supportive role of mentors in helping employees navigate plateaus. This study refines the concept of career plateauing, suggesting that the experience can be more effectively managed through mentorship and reducing the likelihood of negative behaviors like workplace avoidance.

### Practical implications

The practical implications of this study offer valuable insights for HR managers and organizations in managing protégés who experience a career plateau. Mentor proactivity can play a crucial role in understanding and addressing the feelings of plateaued protégés to prevent frustration from escalating into workplace avoidance behavior (Liu et al., 2024). Organizations can implement HR practices that encourage transparent communication and regular check-ins, enabling early detection of career plateauing. By actively engaging with employees, offering growth opportunities, and facilitating role adjustments, mentors can support protégés in overcoming plateaus and mitigating potential negative outcomes (Zeng et al., 2020).

HR practitioners can help organizations tackle plateauing by implementing job redesign and job rotations, which can reinvigorate protégés’ roles and alleviate feelings of stagnation (e.g., Lentz and Allen, 2009; Wang et al., 2014). While hierarchical plateauing may be unavoidable to some extent, alternative strategies, such as involving plateaued protégés in mentorship roles or offering non-promotional rewards, can effectively maintain their motivation and engagement. These strategies foster continued satisfaction and commitment, even in plateaued positions (e.g., Lentz and Allen, 2009). Additionally, organizations can support protégés by offering resources or training on emotional regulation. This is especially important for protégés whose plateau stems from monotonous or unchallenging job content (Opengart and Bierema, 2015). Organizations can help protégés manage frustration constructively and maintain engagement by equipping them with coping strategies and resilience skills. Emotional regulation training, mindfulness programs, or role redesign initiatives can reduce the negative impact of a plateau, promoting both individual well-being and organizational productivity (Lin et al., 2018). These proactive strategies prevent workplace avoidance behavior and contribute to a more resilient and engaged workforce.

### Limitations and future avenues

This study, while contributing valuable insights into the relationships among career plateaus, job frustration, and workplace avoidance behavior, as well as the moderating role of perceived mentor proactivity, has several limitations. First, perceived mentor proactivity was rated by protégés rather than by mentors themselves. These ratings may reflect protégés’ interpretations of mentors’ observable proactive behaviors, relationship quality, and informant-specific biases rather than mentors’ underlying personality traits. Moreover, the construct was assessed only at Time 1 and may not capture changes in protégés’ perceptions as mentoring relationships evolve. Accordingly, the findings should be interpreted as reflecting perceived mentor proactivity rather than an objective assessment of mentor personality. Future research should assess the construct at multiple time points and combine protégé ratings with mentor self-reports, independent evaluations, and behavioral indicators to establish its temporal stability and convergent validity.

Second, the present study is limited by its one-dimensional interpretation of career plateaus, primarily framing them as negative experiences that inevitably lead to job frustration and workplace avoidance. However, this perspective may oversimplify the complexity of how individuals respond to plateaued career trajectories. In reality, some protégés may view career plateaus as opportunities for self-reflection, reskilling, or career redirection, and may remain actively engaged at work while exploring external opportunities. Future research should adopt a more nuanced approach by considering individual differences in how career plateaus are perceived and navigated. This would enhance both the theoretical depth and practical relevance of research in this area.

Third, while the study used a time-lagged design to reduce common method bias, some data, such as career plateauing and job frustration, were self-reported. Self-reports are vulnerable to social desirability bias and self-assessment inaccuracies. Future studies could incorporate objective data or multiple raters to provide a more balanced and accurate picture of the constructs involved. Moreover, while the time-lagged design offers some insights, a longitudinal study with more waves of data collection over a longer period would offer more substantial evidence of causal relationships. This would help determine whether the effects of career plateaus on job frustration and workplace avoidance behavior are persistent or short-lived. Fourth, the study’s focus is on the perceived mentor proactivity as a boundary condition. Other characteristics of mentors, such as emotional intelligence, leadership style, and specific mentoring behaviors, could also play a significant role in alleviating the effects of career plateaus. Exploring a broader range of mentor traits in future research could provide a more nuanced understanding of the mentor’s role in this dynamic.

## Conclusion

This study provides a theoretical framework highlighting the significant impact of protégés career plateaus on job frustration and workplace avoidance behavior. By drawing on affective event theory, we demonstrate how the emotional experiences of plateaued employees, triggered by career stagnation, can lead to job frustration and workplace avoidance behavior. Importantly, we asserted that a perceived mentor proactivity could be crucial in buffering these effects, influencing the direct relationship between protégés’ career plateaus and job frustration and the subsequent indirect effects on workplace avoidance behavior. While career plateaus are often perceived as detrimental to employees and organizations, they do not necessarily lead to negative outcomes if managed effectively. Perceived mentor proactivity can intervene to mitigate the negative emotional consequences of career stagnation, offering valuable support to plateaued protégés and helping them navigate these challenging career phases. This highlights the importance of mentor-mentee relationships in maintaining employee engagement and preventing disengagement-related behaviors in the workplace.

## Data Availability

The raw data supporting the conclusions of this article will be made available by the authors, without undue reservation.
